# Emotional Inertia is Associated with Lower Well-Being when Controlling for Differences in Emotional Context

**DOI:** 10.3389/fpsyg.2015.01997

**Published:** 2016-01-08

**Authors:** Peter Koval, Stefan Sütterlin, Peter Kuppens

**Affiliations:** ^1^School of Psychology, Australian Catholic University, FitzroyVIC, Australia; ^2^Research Group of Quantitative Psychology and Individual Differences, Faculty of Psychology and Educational Sciences, KU LeuvenLeuven, Belgium; ^3^Section of Psychology, Department of Social Science, Lillehammer University CollegeLillehammer, Norway; ^4^Department of Psychosomatic Medicine, Division of Surgery and Clinical Neuroscience, Oslo University Hospital – RikshospitaletOslo, Norway

**Keywords:** emotion dynamics, affect dynamics, emotional inertia, well-being, positive emotions, negative emotions, film-clips, emotional context

## Abstract

Previous studies have linked higher emotional inertia (i.e., a stronger autoregressive slope of emotions) with lower well-being. We aimed to replicate these findings, while extending upon previous research by addressing a number of unresolved issues and controlling for potential confounds. Specifically, we report results from two studies (*N*s = 100 and 202) examining how emotional inertia, assessed in response to a standardized sequence of emotional stimuli in the lab, correlates with several measures of well-being. The current studies build on previous research by examining how inertia of both positive emotions (PE) and negative emotions (NE) relates to positive (e.g., life satisfaction) and negative (e.g., depressive symptoms) indicators of well-being, while controlling for between-person differences in the mean level and variability of emotions. Our findings replicated previous research and further revealed that (a) NE inertia was more strongly associated with lower well-being than PE inertia; (b) emotional inertia correlated more consistently with negative indicators (e.g., depressive symptoms) than positive indicators (e.g., life satisfaction) of well-being; and (c) these relationships were independent of individual differences in mean level and variability of emotions. We conclude, in line with recent findings, that higher emotional inertia, particularly of NE, may be an indicator of increased vulnerability to depression.

## Introduction

Emotions are not static, but are rather characterized by frequent ups and downs (Frijda, 2007; Scherer, 2009). Such emotional fluctuations can be thought of as the outputs of an affective system that responds to external events and internal regulatory processes (Larsen, 2000; Kuppens et al., 2010b). A consensus is now emerging that individual differences in these patterns of emotional ups and downs, referred to as *affect dynamics*^1^, are important markers of psychological functioning and well-being (Davidson, 1998, 2015; Hollenstein et al., 2013; Houben et al., 2015; Trull et al., 2015; Wichers et al., 2015). Thus, in addition to studying mean levels of emotion (i.e., trait affect), charting the patterns with which affective states fluctuate and change over time may offer novel insights into emotional functioning.

### Emotional Inertia and Well-Being

Here, we focus on a pattern of affect dynamics labeled *emotional inertia*, as captured by the autocorrelation of an emotional trajectory over time. The autocorrelation of an emotion indicates how strongly emotional intensity at a given time-point predicts emotional intensity at the next time-point. Thus, higher autocorrelations (i.e., higher inertia) reflect emotions that are more self-predictable or resistant to change over time (Cook et al., 1995; Suls et al., 1998; Kuppens et al., 2010a). Given that a central function of emotions is to change in response to events and regulatory efforts (Larsen, 2000; Frijda, 2007; Kuppens et al., 2010b), higher levels of inertia may indicate that emotions have become decoupled from internal and/or external contingencies, and may have thus become dysfunctional (Kuppens et al., 2010a; Butler, 2011; Hollenstein et al., 2013). Put otherwise, a high degree of emotional inertia may reflect a lack of emotional flexibility–the capacity to continually adapt emotional responding to fluctuating situational and regulatory demands–which is considered essential to psychological health and well-being (Kashdan and Rottenberg, 2010; Waugh et al., 2011; Hollenstein et al., 2013; Hollenstein, 2015). We define well-being, following Houben et al. (2015), as a broad construct comprising lower levels of maladjustment [e.g., depressive symptoms, negative affectivity (NA)] or psychological vulnerability (e.g., neuroticism, habitual rumination) as well as higher levels of flourishing [e.g., positive affectivity (PA), satisfaction with life] or resilience (e.g., self-esteem, extraversion).^2^ Given the ubiquity of emotions in human functioning, emotional flexibility would be expected to relate to a wide range of well-being measures. This view is supported by a growing number of studies linking higher emotional inertia with various indicators of maladjustment, including neuroticism (Suls et al., 1998), low self-esteem (Kuppens et al., 2010a), impaired social functioning (Fairbairn and Sayette, 2013), habitual rumination (Koval et al., 2012; Brose et al., 2014), elevated depressive symptoms (Wenze et al., 2009; Koval and Kuppens, 2012; Koval et al., 2012, 2013; Brose et al., 2014), and increased risk of Major Depressive Disorder (Kuppens et al., 2012; van de Leemput et al., 2014).

### Open Questions Regarding the Association Between Emotional Inertia and Well-Being

#### Endogenous versus Exogenous Influences

Although previous research has consistently linked emotional inertia with lower well-being (Houben et al., 2015) a number of important issues remain unresolved. First, research on emotional inertia (especially inertia of subjective feelings) has relied heavily on experience sampling or ecological momentary assessment methods to study emotional fluctuations in daily life (Houben et al., 2015). Although studying affect dynamics using naturalistic methods has several advantages (e.g., high ecological validity), a major shortcoming of this approach is a lack of control over contextual factors. As a result, it is unclear whether individual differences in emotional inertia in daily life are due to endogenous (e.g., emotional reactivity and regulation) versus exogenous (e.g., differential exposure to events) factors, or both. Indeed, recent evidence suggests that higher inertia of negative emotions in daily life is at least partly driven by exposure to more intense negative events (Koval et al., 2015a). However, the only way to conclusively demonstrate that heightened inertia is due to processes endogenous to the individual, and therefore reflects inflexible emotional responding, is to expose all individuals to the same emotional events in the same order. We have previously assessed emotional inertia in response to a standardized sequence of stimuli and found a positive association between inertia of negative emotions (NE) and depressive symptoms, supporting the view that endogenously caused emotional inflexibility is related to lower well-being (Koval et al., 2013). However, in Koval et al. (2013), we calculated emotional inertia separately for each individual using the within-person autocorrelation based on only 11 occasions. In contrast, other studies have typically estimated emotional inertia simultaneously for all individuals using a multilevel autoregressive model, and using many more time-points (e.g., Suls et al., 1998; Kuppens et al., 2010a; Koval et al., 2012). Both the small number of time-points and the use of a two-step analytic approach can undermine the reliability of results (Wang et al., 2012). We address these limitations in the current studies by increasing the number of time-points used to model inertia and using multilevel autoregressive models rather than calculating autocorrelations separately for each individual.

#### Inertia of Positive Emotions

Second, it remains unclear under which circumstances higher inertia of positive emotions (PE) is maladaptive. Theoretically, emotional flexibility is adaptive independent of valence (Kashdan and Rottenberg, 2010; Hollenstein et al., 2013). Indeed, PE that are slow to change across contexts may also indicate an affective system that has become disconnected from environmental contingencies or regulatory processes (Kuppens et al., 2010a; Gruber, 2011; Kuppens et al., 2012). In line with this, Houben et al.’s (2015) meta-analysis found that higher inertia of PE was consistently related to lower well-being, although to a lesser extent than NE inertia. However, this finding may be driven by studies examining inertia of positive emotional behaviors assessed at a short timescale in the lab (Gottman et al., 2002; Kuppens et al., 2012; Fairbairn and Sayette, 2013). In contrast, research looking at inertia of positive feelings, typically assessed at longer timescales and in daily life using experience sampling, has produced less consistent results (Kuppens et al., 2010a; Höhn et al., 2013; Koval et al., 2013). It is important to establish whether these divergent findings are due to methodological differences (i.e., short vs. long timescale; lab vs. daily life) or because of differences in the dynamics of behavioral versus experiential components of emotions (see also, Koval et al., 2015b). Studies assessing inertia of positive feelings at short timescales (i.e., seconds or minutes) in the lab are needed to answer this question.

#### Positive Indicators of Well-Being

Third, research on emotional inertia has mostly focused on its associations with indicators of maladjustment (i.e., negative indicators of well-being), such as neuroticism (Suls et al., 1998), rumination (Koval et al., 2012), depressive symptoms (Koval et al., 2013), and MDD diagnosis (Kuppens et al., 2012). In contrast, very few studies have examined how emotional inertia relates to flourishing (i.e., positive indicators of well-being) such as satisfaction with life, trait PA, or extraversion (Diener et al., 1999). One exception, which has been associated with lower inertia, is self-esteem (Kuppens et al., 2010a; Koval and Kuppens, 2012). However, the overall bias in the literature on emotional inertia toward indicators of maladjustment is reflected in Houben et al.’s (2015) meta-analysis, which reported more than twice as many effect sizes linking emotional inertia with negative than positive indicators of well-being. Redressing this imbalance would help to determine whether higher inertia should be considered an indicator of well-being in general, or rather only a marker of maladjustment.

#### Emotional Inertia versus Emotional Variability

Finally, previous studies linking emotional inertia with lower well-being have typically not controlled for individual differences in the variability (e.g., within-person *SD*) of emotions (but see, Koval et al., 2013). Although these two measures are distinct–variability represents the overall range of an emotion over time (regardless of the temporal order of emotional intensity levels), whereas emotional inertia reflects the moment-to-moment predictability or temporal dependency of emotions–they are mathematically and empirically related (Wang et al., 2012; Koval et al., 2013). Further complicating matters, inertia and variability are sometimes combined into an overall index of emotional instability, known as the mean square successive difference (MSSD; Jahng et al., 2008; Wang et al., 2012). Importantly, both greater variability (*SD*) and instability (*MSSD*) of emotions have also been associated with lower well-being (Houben et al., 2015). Thus, it remains unclear whether the negative association between emotional inertia and well-being reported in previous studies is (partly) driven by individual differences in variability. Similarly, given that trait levels of positive and negative emotions are known to correlate with other indicators of well-being and psychological functioning (e.g., Watson et al., 1988a), it is important to control for individual differences in mean level of emotions when studying patterns of emotion dynamics, such as emotional inertia (Trull et al., 2015).

### The Present Studies

The current studies aimed to replicate and extend upon previous research on the association between emotional inertia (of subjective feelings) and well-being in two samples (*N*s = 100 and 202) and meta-analyze findings across studies to arrive at robust conclusions. Since most previous studies on the inertia of feelings have relied on experience sampling, our first aim was to assess emotional inertia using a validated film-task administered in the lab. Second, we aimed to examine how the inertia of negative and positive feelings is related to both positive (e.g., life satisfaction) and negative (e.g., depressive symptoms) indicators of well-being. Finally, because the association between emotional inertia and well-being may be partly driven by mean levels and variability (i.e., *SD*) of emotions (Jahng et al., 2008; Wang et al., 2012; Koval et al., 2013), we modeled emotional inertia using both raw (unstandardized) and within-person standardized emotion ratings. The latter approach controls for between-person differences in mean level and variability of emotions (Koval et al., 2013). In line with previous theory and research, we predicted that higher inertia of both NE and PE would be related to lower well-being. Put otherwise, we expected inertia to be negatively associated with positive indicators of well-being and positively associated with negative indicators of well-being. Finally, based on our previous findings (Koval et al., 2013) we predicted that even after removing between-person differences in mean level and variability of emotions, greater emotional inertia would continue to be associated with lower well-being.

## Materials and Methods

### Ethics Statement

Both studies were approved by the ethics committee of the Faculty of Psychology and Educational Sciences, KU Leuven. All participants provided written informed consent.

### Participants

#### Study 1

One hundred students (86% female), aged between 18 and 28 years (*M* = 20.77; *SD* = 2.15), were recruited from a large database of volunteers maintained by the psychology department at the KU Leuven and by advertising around university buildings. Participants were reimbursed €8 for their time.

#### Study 2

As part of a broader three-wave longitudinal study on emotional functioning, we aimed to recruit 200 students commencing their first year of tertiary education. We advertised at secondary schools and tertiary education orientation/information sessions in the Leuven area. To maximize variability in well-being, we recruited an initial pool of 686 students (65.7% female) to complete an online pre-screening using the Center for Epidemiologic Studies Depression Scale (CES-D; Radloff, 1977) and, using a stratified sampling approach (Ingram and Siegle, 2009), we invited an equal number of participants from each quintile of the CES-D range to participate in the study. However, this strategy was only partially successful: we were able to recruit 180 participants with a relatively broad range of CES-D pre-screening scores (Range = 0–39; *M* = 14.41, *SD* = 8.41). To achieve our original target sample size, we recruited an additional 22 participants after the study had already begun, who did not complete the CES-D pre-screening. At the time of the study, these additional 22 participants did not differ significantly on the CES-D (*M* = 11.86, *SD* = 7.11) from the remainder of the sample (*M* = 12.55, *SD* = 7.80), *t*(200) = 0.393, *p* = 0.695. The final sample comprised 202 students (55% female) aged between 17 and 24 years (*M* = 18.32, *SD* = 0.96). Participants were eligible to receive up to 60€ for their completion of all tasks in each wave of the longitudinal study, plus an additional bonus of 60€ for completing all three waves.

### Procedure

In both Studies 1 and 2, participants attended the lab in small groups and were individually seated in separate cubicles where they completed a number of measures including the well-being questionnaires and emotional film-task, described below. Other measures not directly relevant to the current investigation and not reported here, were in Study 1: (a) resting heart rate, (b) heart-beat counting task, and (c) several other self-report questionnaires; and in Study 2: (a) structured clinical interviews; (b) additional self-report questionnaires; (c) lab tasks assessing cognitive and emotional functioning; and d) a 7-day experience sampling protocol. No other measures or manipulations were administered.

### Well-Being Measures

Our selection of specific well-being measures was guided by (i) a desire to represent a range of both positive indicators of well-being (i.e., measures of flourishing, or psychological resilience) and negative indicators of well-being (i.e., measures of maladjustment, or psychological vulnerability), and (ii) the availability of identical (or highly similar) measures across both studies to allow for synthesis of findings using meta-analysis. For each well-being measure, we calculated mean scores (after reverse-coding items, where required). Descriptive statistics and correlations among all well-being measures are shown in **Table 1**.

**Table 1:** Descriptive statistics and correlations among well-being measures.

	Study 1 (*N* = 100)				Study 2 (*N* = 202)											

	Range				Range				Correlations							
Well-being measure	Possible	Actual	*M*	*SD*	Possible	Actual	*M*	*SD*	*SWLS*	*RSE*	*PA*	*E*	*CESD*	*RRS*	*NA*	*N*
Life Satisfaction (SWLS)	1–7	2.6–7.0	5.20	0.99	1–7	2.4–6.8	5.17	1.01		**0.66**	**0.42**	**0.25**	**–0.55**	**–0.39**	**–0.31**	**–0.35**
Self-Esteem (RSE)	1–4	1.8–4.0	3.30	0.51	1–4	1.2–4.0	3.08	0.58	**0.61**		**0.45**	**0.31**	**–0.61**	**–0.53**	**–0.43**	**–0.48**
Positive Affectivity (PA)	1–5	2.3–4.5	3.42	0.51	1–5	2.0–4.7	3.34	0.52	**0.37**	**0.51**		**0.39**	**–0.42**	**–0.27**	–0.12	**–0.26**
Extraversion (E)	1–5	2.4–5.0	3.59	0.56	1–7	1.5–7.0	4.87	1.31	**0.39**	0.29	**0.44**		**–0.22**	**–0.24**	–0.20	–0.07
Depression (CESD)	0–3	0.0–1.5	0.55	0.39	0–3	0.0–1.9	0.62	0.39	**–0.55**	**–0.56**	–0.28	**–0.33**		**0.58**	**0.50**	**0.51**
Rumination (RRS)	1–4	1.1–3.2	1.79	0.45	1–4	1.0–3.2	1.90	0.46	**–0.34**	**–0.34**	–0.09	–0.14	**0.53**		**0.48**	**0.45**
Negative Affectivity (NA)	1–5	1.0–3.4	1.84	0.60	1–5	1.0–4.2	1.98	0.55	**–0.46**	**–0.49**	–0.07	–0.19	**0.63**	**0.38**		**0.59**
Neuroticism (N)	1–5	1.1–4.4	2.94	0.70	1–7	1.0–7.0	3.27	1.33	**–0.31**	**–0.52**	**–0.35**	–0.08	**0.58**	**0.35**	**0.62**	

*Correlations between well-being measures in Study 1 (*N* = 100) are shown below the diagonal. Correlations between well-being measures in Study 2 (*N* = 202) are shown above the diagonal. Correlations shown in **bold** are statistically significant at *p* < 0.001.*

#### Positive Indicators of Well-Being

##### Life satisfaction

Participants’ life satisfaction was assessed using the Satisfaction with Life Scale (SWLS; Diener et al., 1985). The SWLS is widely used to assess the cognitive component of subjective well-being (Diener et al., 1999). Participants are asked to rate their agreement with five items regarding global evaluations of their life (e.g., “in most ways my life is close to my ideal”) on a scale from 1 (*strongly disagree*) to 7 (*strongly agree*). Cronbach’s α was 0.83 in both studies.

##### Self-esteem

Global self-esteem, which reflects a person’s satisfaction with him/herself, is considered to be a component of subjective well-being, at least in individualist cultures (Diener et al., 1999). Self-esteem has also been related to improved mental and physical health (Taylor and Brown, 1988; Mann et al., 2004). Participants completed the Rosenberg Self-Esteem questionnaire (RSE; Rosenberg, 1989), comprising 10 items (e.g., “on the whole, I am satisfied with myself”) that assess global self-esteem. Responses are on a scale from 1 (*strongly disagree*) to 4 (*strongly agree*). Cronbach’s αs were 0.87 and 0.90 in Studies 1 and 2, respectively.

##### Positive affectivity

Trait PA, reflecting the tendency to experience frequent and intense positive feelings in daily life, was assessed with the Positive and Negative Affect Schedule (Watson et al., 1988b). Participants rated how much they generally experience 10 high-activation positive (e.g., “interested,” “excited”) feelings during their normal daily lives using a scale from 1 (*very slightly or not at all*) to 5 (*extremely*). Cronbach’s αs were 0.78 and 0.81 in Studies 1 and 2, respectively.

##### Extraversion

Extraversion has been related to higher levels of happiness and well-being (Costa and McCrae, 1980; DeNeve and Cooper, 1998; Gale et al., 2013) and lower levels of psychopathology (Trull and Sher, 1994). In Study 1, participants completed the extraversion (E) subscale of the Big Five Inventory (John et al., 2008), comprising eight items rated on a scale from 1 (*strongly disagree*) to 5 (*strongly agree*). In Study 2, participants completed the extraversion subscale of the Ten Item Personality Inventory (Gosling et al., 2003), comprising two items rated on a scale from 1 (*strongly disagree*) to 7 (*strongly agree*). Cronbach’s αs were 0.78 and 0.62 in Studies 1 and 2, respectively.

#### Negative Indicators of Well-Being

##### Depressive symptoms

Participants completed the CES-D (Radloff, 1977), a 20-item questionnaire assessing the frequency of depressive symptoms (e.g., “I had crying spells”; “I felt depressed”) over the past week on a scale from 0 (*rarely or none of the time*) to 3 (*most or all of the time*). Elevated scores on the CES-D are indicative of distress and poor well-being, and predict future clinical depression diagnosis independent of other known risk factors (Klein et al., 2013). Cronbach’s αs were 0.89 and 0.88 in Studies 1 and 2, respectively.

##### Rumination

Trait rumination is related to poor well-being, and increased vulnerability for many psychopathologies, including depression, anxiety, substance abuse, and eating disorders (Nolen-Hoeksema et al., 2008). Participants completed the Ruminative Responses Scale (RRS; Treynor et al., 2003), a 22-item measure of the tendency to habitually ruminate (e.g., to think “Why can’t I handle things better?”) in response to episodes of sad or depressed mood. Responses were on scale from 1 (*almost never*) to 4 (*almost always*). Cronbach’s αs were 0.88 and 0.90 in Studies 1 and 2, respectively.

##### Negative affectivity

Trait NA, reflecting the tendency to experience frequent and intense negative feelings in daily life, was assessed with the Positive and Negative Affect Schedule (Watson et al., 1988b). Participants rated how much they generally experience 10 high-activation negative (e.g., “irritable” “upset”) feelings during their normal daily lives on a scale from 1 (*very slightly or not at all*) to 5 (*extremely*). Cronbach’s αs were 0.86 and 0.84 in Studies 1 and 2, respectively.

##### Neuroticism

In Study 1, participants completed the neuroticism (N) subscale of the Big Five Inventory (John et al., 2008), comprising eight items rated on a scale from 1 (*strongly disagree*) to 5 (*strongly agree*). In Study 2, neuroticism was assessed with the two-item neuroticism subscale of the Ten Item Personality Inventory (Gosling et al., 2003), rated on a scale from 1 (*strongly disagree*) to 7 (*strongly agree*). Neuroticism is a major risk factor for poor mental and physical health (Lahey, 2009) and is associated with lower levels of well-being (Costa and McCrae, 1980; DeNeve and Cooper, 1998; Gale et al., 2013) Cronbach’s αs were 0.83 and 0.59 in Studies 1 and 2, respectively.

### Emotional Film-Task

This task was a slightly modified version of the task described in Koval et al. (2013). The task was programmed and administered using E-prime (Psychology Software Tools, Inc.). Participants watched 10 emotional film-clips (four negative, four positive, and two neutral), shown in a fixed order, and rated their subjective experiences of PE and NE following each film. To ensure ratings reflected momentary emotional responses to the films, participants were instructed to rate their current feelings rather than how they felt that day or in general. To reduce demand effects, participants were encouraged to rate their emotions honestly and intuitively, rather than in terms of how they thought a person *should* feel after seeing each film-clip. These instructions were reinforced by imposing a 10 s time limit for completing each emotion rating (reduced to 5 s in Study 2 due to time limitations). We adapted the original task by adding a 30 s “rest” after each film-clip (reduced to 20 s in Study 2 due to time restrictions). During rest, participants were asked to keep their attention on the screen (displaying a neutral image of a ball of colored string), and subsequently rated their subjective feelings. Thus, participants rated their feelings of PE and NE on 21 occasions (at baseline, following each of the 10 films, following each of the 10 rests). At each occasion, participants rated how *sad, angry, depressed, anxious, relaxed*, and *happy* they felt on a scale from 0 (*not at all*) to 6 (*very much*). The task began with a practice trial (participants watched a neutral film-clip and completed ratings of PE and NE), which was not included in the analyses. Ratings on the four negative items and two positive items were averaged to form NE and PE scales, respectively. Following Nezlek (2012), we estimated within-person reliabilities of the NE and PE scales using three-level unconditional models. NE reliabilities were 0.76 (Study 1) and 0.80 (Study 2). PE reliabilities were 0.62 (Study 1) and 0.76 (Study 2).

### Data Analyses

Given that participants repeatedly rated their emotions in response to the Emotional Film-Task, the resulting data can be thought of as nested or multilevel, with measurement occasions (Level-1) nested within participants (Level-2). To account for the resulting non-independence and provide appropriate estimates of standard errors, we analyzed data using multilevel modeling (Bolger and Laurenceau, 2013). Specifically, we ran two-level autoregressive (AR1) models, including both a random intercept and a random (autoregressive) slope in each model. We conducted separate analyses for PE and NE, and separately using raw and within-person standardized emotion ratings. Separate analyses examined how each measure of well-being correlated with individual differences in inertia of PE or NE. Further details of these models, including model equations, are provided below. All multilevel analyses were conducted using HLM 7.01 (Raudenbush et al., 2013).

#### Raw Emotion Ratings

In line with previous research on emotional inertia (e.g., Suls et al., 1998; Kuppens et al., 2010a), we used multilevel modeling to estimate associations between well-being and emotional inertia, based on the raw emotion scores obtained from the film-task. At Level-1, we modeled the first-order autoregressive slope of emotions (representing emotional inertia), as shown in Eq. 1, below:

**Level-1 (Occasions):**

(1) $${\mathrm{Emotion}}_{ti}=\;\;\pi_{0i}\;+\;\;\pi_{1i}({\mathrm{Emotion}}_{t-1i})+\;e_{ti}$$

Here, the outcome at Level-1 (Emotion*_ti_*) represents person *i*’s level of emotion at time *t*. The autoregressive slope (π_1_*_i_*) reflects how strongly person *i*’s level of emotion at time *t* is related to their level of emotion at time *t* – *1*, the previous occasion. This autoregressive slope is a direct operationalization of emotional inertia (e.g., Suls et al., 1998). The lagged predictor (Emotion*_t__-__1i_*) was person-mean centered to remove between-person differences from Level-1 parameter estimates (Enders and Tofighi, 2007).^3^ As a result, the Level-1 intercept (π_0_*_i_*) is practically equal to each person *i*’s mean level of emotion across all occasions. The Level-1 intercept and slope were allowed to vary randomly across persons at Level-2, and their associations with well-being were modeled, as shown in Eqs 2 and 3, below.

**Level-2 (Persons):**

(2) $$\pi_{0i}\;=\;\;\beta_{00}+\;\;\beta_{01}(z{Well-Being}_{i})\;+\;r_{0i}$$

(3) $$\pi_{1i}\;=\;\;\beta_{10}+\;\;\beta_{11}(z{Well-Being}_{i})+r_{1i}$$

Well-being measures were converted to *z*-scores before being entered at Level-2. Thus, of direct relevance to this report, the Level-2 intercept (β_10_) reflects the average level of emotional inertia across the sample, and the Level-2 slope (β_11_) reflects the association between well-being and emotional inertia. These β_11_ values are displayed in **Tables 3** and **4**, and can be interpreted as standardized regression weights. For instance, if β_11_ = 0.10, an individual scoring 1 *SD* above the sample-mean on the measure of well-being is predicted to have an emotional inertia level 0.10 units higher than the sample average, whereas a person scoring 1 *SD* below the sample-mean on well-being is predicted to have an emotional inertia level 0.10 units lower than average. Note that the autoregressive slope reflecting emotional inertia is comparable to an autocorrelation and typically ranges between 0 and 1 (Hamaker, 2012).

#### Within-Person Standardized Emotion Ratings

Our previous research has demonstrated the importance of controlling for individual differences in mean level and variability of emotions when examining associations between emotional inertia and well-being (Koval et al., 2013). We therefore replicated all analyses using within-person standardized emotion ratings. Emotion scores were standardized per individual by subtracting each person’s mean emotion rating (across all occasions) from their emotion rating at each occasion, and then dividing by the *SD* of the person’s emotion ratings (across all occasions), as shown in Eq. 4, below.^4^

(4) $$z{\mathrm{Emotion}}_{ti}\;=\;\frac{{\mathrm{Emotion}}_{ti}-{\bar{\mathrm{Emotion}}}_{i}}{SD{\left(\mathrm{Emotion}\right)}_{i}}$$

These standardized emotion scores were analyzed using multilevel models identical to those shown in Eqs 1–3, with the exception that the lagged predictor was not person-mean centered. Person-mean centering is not required because the within-person standardized emotion ratings have already removed all between-person differences in mean level and variability.

#### Meta-Analyses

To identify consistent findings across studies, we meta-analyzed results from Studies 1 and 2 using Comprehensive Meta-Analysis (CMA; Borenstein et al., 2005). Following the approach used by (Houben et al., 2015), estimates obtained from multilevel models were converted to Pearson correlations based on their *p*-values and degrees of freedom (Lipsey and Wilson, 2001). We then computed the average effect size (i.e., correlation) across Studies 1 and 2 using random-effects models in CMA.

## Results

### Preliminary Analyses: Average Levels of Emotional Inertia

We estimated average levels of NE and PE inertia in each sample using multilevel models identical to those described above, but with no Level-2 predictors. Following Hamaker and Grasman (2015), lagged predictors were entered uncentered in these initial analyses to obtain unbiased estimates of average emotional inertia levels in each sample. As shown in **Table 2**, across both studies autoregressive slopes were positive (0.151 ≤ β_10_ ≤ 0.397) and statistically significant (*p*s < 0.001) indicating that both PE and NE showed significant moment-to-moment predictability.

**Table 2:** Results of multilevel autoregressive models estimating average levels of emotional inertia.

	Study 1 (*N* = 100)				Study 2 (*N* = 202)			
	Fixed effect (β_10_)			Random effect (*r*_1_)	Fixed effect (β_10_)			Random effect (*r*_1_)
Model	Est. (*SE*)	95% CI		*SD*	Est. (*SE*)	95% CI		*SD*
		**LL**	**UL**			**LL**	**UL**	
NE inertia (Raw)	0.40 (0.02)	0.35	0.45	0.10	0.33 (0.02)	0.29	0.36	0.11
NE inertia (Standardized)^a^	0.25 (0.03)	0.20	0.30	0.14	0.22 (0.02)	0.19	0.25	0.06
PE inertia (Raw)	0.31 (0.02)	0.26	0.36	0.09	0.25 (0.02)	0.22	0.29	0.09
PE inertia (Standardized)	0.22 (0.02)	0.17	0.26	0.06	0.15 (0.01)	0.12	0.18	0.02

*Fixed effect estimates representing average levels of emotional inertia are significantly different from zero at *p* < 0.001.*

*^a^*n* = 98 for analyses using standardized NE scores in Study 1.*

### Associations Between Well-Being and Emotional Inertia

Results of multilevel models estimating associations between NE and PE inertia with positive and negative indicators of well-being are shown in **Tables 3** and **4**, respectively. Given the large number of results, we focus our discussion on findings that were consistent across studies and analytic methods (i.e., raw vs. standardized emotion ratings).

**Table 3:** Results of multilevel autoregressive models estimating associations between emotional inertia and positive indicators of well-being.

	Study 1 (*N* = 100)^a^				Study 2 (*N* = 202)				Meta-analysis			
		95% CI				95% CI				95% CI		
Well-being measure	Est. (*SE*)	*LL*	*UL*	*p*	Est. (*SE*)	*LL*	*UL*	*p*	*r*	*LL*	*UL*	*p*
**Life satisfaction**												
NE inertia (Raw)	-0.01 (0.03)	–0.07	0.04	0.644	-0.04 (0.02)	–0.08	-0.01	0.014	–0.13	–0.24	–0.01	0.030
NE inertia (Standardized)	-0.02 (0.03)	–0.07	0.03	0.505	-0.03 (0.02)	–0.06	0.00	0.061	–0.11	–0.22	0.00	0.055
PE inertia (Raw)	-0.01 (0.02)	–0.06	0.03	0.595	-0.02 (0.02)	–0.05	0.02	0.383	–0.06	–0.17	0.05	0.309
PE inertia (Standardized)	0.00 (0.02)	–0.04	0.04	0.948	-0.01 (0.02)	–0.04	0.02	0.341	–0.04	–0.16	0.07	0.458
**Self-esteem**												
NE inertia (Raw)	-0.03 (0.02)	–0.08	0.02	0.187	-0.03 (0.02)	–0.06	0.00	0.050	–0.14	–0.25	–0.02	0.018
NE inertia (Standardized)	-0.06 (0.02)	–0.11	–0.01	0.015	-0.04 (0.01)	–0.07	-0.01	0.011	–0.20	–0.31	–0.09	<0.001
PE inertia (Raw)	-0.03 (0.02)	–0.08	0.01	0.124	-0.03 (0.02)	–0.06	0.00	0.074	–0.14	–0.25	–0.02	0.019
PE inertia (Standardized)	-0.02 (0.02)	–0.06	0.02	0.354	-0.02 (0.01)	–0.04	0.01	0.243	–0.09	–0.20	0.03	0.137
**Positive affectivity**												
NE inertia (Raw)	-0.01 (0.02)	–0.05	0.03	0.752	-0.02 (0.02)	–0.05	0.01	0.252	–0.06	–0.18	0.05	0.263
NE inertia (Standardized)	-0.03 (0.03)	–0.09	0.03	0.327	0.00 (0.01)	–0.03	0.02	0.746	–0.02	–0.13	0.10	0.768
PE inertia (Raw)	0.01 (0.02)	–0.03	0.05	0.724	-0.02 (0.02)	–0.05	0.01	0.250	–0.04	–0.16	0.07	0.459
PE inertia (Standardized)	0.00 (0.02)	–0.04	0.04	0.891	-0.03 (0.01)	–0.06	0.00	0.024	–0.09	–0.25	0.08	0.303
**Extraversion**												
NE inertia (Raw)	-0.04 (0.02)	–0.09	0.01	0.084	0.00 (0.01)	–0.03	0.02	0.827	–0.07	–0.25	0.12	0.484
NE inertia (Standardized)	-0.07 (0.03)	–0.12	–0.01	0.015	0.01 (0.01)	–0.02	0.03	0.711	–0.10	–0.36	0.17	0.459
PE inertia (Raw)	0.02 (0.02)	–0.03	0.07	0.395	0.01 (0.02)	–0.03	0.05	0.642	0.05	–0.06	0.16	0.386
PE inertia (Standardized)	0.01 (0.02)	–0.03	0.06	0.603	-0.01 (0.02)	–0.04	0.02	0.686	0.00	–0.12	0.11	0.973

*Meta-analysis results (based on random-effects models) show the average effect size, *r*, across Studies 1 and 2.*

*^a^*n* = 98 for all analyses using standardized negative emotion scores in Study 1.*

**Table 4:** Results of multilevel autoregressive models estimating associations between emotional inertia and negative indicators of well-being.

	Study 1 (*N* = 100)^a^				Study 2 (*N* = 202)				Meta-Analysis			
		95% CI				95% CI				95% CI		
Well-Being Measure	Est. (*SE*)	*LL*	*UL*	*p*	Est. (*SE*)	*LL*	*UL*	*p*	*r*	*LL*	*UL*	*p*
**Depressive symptoms**												
NE inertia (Raw)	0.05 (0.03)	0.00	0.11	0.063	0.04 (0.02)	0.01	0.07	0.016	0.18	0.06	0.28	0.002
NE inertia (Standardized)	0.08 (0.03)	0.03	0.13	0.004	0.03 (0.02)	0.00	0.07	0.029	0.20	0.07	0.33	0.002
PE inertia (Raw)	0.04 (0.02)	-0.01	0.09	0.115	0.03 (0.02)	-0.01	0.06	0.177	0.12	0.00	0.23	0.045
PE inertia (Standardized)	0.03 (0.02)	-0.02	0.08	0.181	0.02 (0.01)	-0.01	0.05	0.128	0.12	0.00	0.23	0.044
**Rumination**												
NE inertia (Raw)	0.05 (0.02)	0.00	0.10	0.043	0.04 (0.02)	0.01	0.07	0.007	0.19	0.08	0.30	0.001
NE inertia (Standardized)	0.07 (0.02)	0.02	0.11	0.006	0.05 (0.01)	0.02	0.08	<0.001	0.26	0.15	0.36	<0.001
PE inertia (Raw)	0.04 (0.02)	-0.01	0.08	0.109	0.04 (0.02)	0.01	0.07	0.010	0.17	0.06	0.28	0.002
PE inertia (Standardized)	0.02 (0.02)	-0.03	0.07	0.442	0.04 (0.01)	0.01	0.07	0.004	0.16	0.04	0.27	0.007
**Negative affectivity**												
NE inertia (Raw)	0.06 (0.02)	0.02	0.10	0.003	0.03 (0.02)	0.00	0.06	0.085	0.20	0.02	0.36	0.027
NE inertia (Standardized)	0.07 (0.02)	0.03	0.11	0.002	0.04 (0.02)	0.00	0.07	0.031	0.21	0.06	0.36	0.006
PE inertia (Raw)	0.03 (0.02)	-0.01	0.07	0.168	0.04 (0.02)	0.01	0.08	0.006	0.18	0.06	0.28	0.002
PE inertia (Standardized)	0.02 (0.02)	-0.02	0.06	0.381	0.03 (0.01)	0.00	0.06	0.028	0.13	0.02	0.24	0.021
**Neuroticism**												
NE inertia (Raw)	0.04 (0.03)	-0.02	0.09	0.166	0.04 (0.02)	0.00	0.07	0.045	0.14	0.03	0.25	0.015
NE inertia (Standardized)	0.08 (0.02)	0.03	0.12	0.002	0.03 (0.02)	0.00	0.07	0.046	0.21	0.04	0.37	0.014
PE inertia (Raw)	0.03 (0.02)	-0.02	0.07	0.228	0.04 (0.02)	0.00	0.07	0.037	0.14	0.03	0.25	0.016
PE inertia (Standardized)	0.03 (0.02)	-0.02	0.07	0.249	0.03 (0.01)	0.00	0.05	0.051	0.13	0.02	0.24	0.024

*Meta-analysis results (based on random-effects models) show the average effect size, *r*, across Studies 1 and 2.*

*^a^*n* = 98 for all analyses using standardized negative emotion scores in Study 1.*

### Positive Indicators of Well-Being

Of the positive indicators of well-being included in the current studies, only self-esteem showed consistent associations with emotional inertia across studies and analytic methods (see meta-analysis in **Table 3**). Replicating previous findings, self-esteem was negatively associated with NE inertia (Houben et al., 2015). However, whereas previous studies (e.g., Kuppens et al., 2010a) have also reported a negative association between self-esteem and inertia of PE, the evidence for such an association was weaker in the current studies. In particular, when PE scores were standardized there was no longer a significant association between PE inertia and self-esteem. Thus, while individuals with higher self-esteem clearly showed lower levels of NE inertia, the association between self-esteem and PE inertia was weaker and appeared to be partly accounted for by individual differences in mean level or variability of emotions. These findings are broadly consistent with Houben et al.’s (2015) meta-analysis, which found that self-esteem was more strongly related to the inertia of NE than PE. However, the current studies extend upon Houben et al.’s (2015) synthesis of previous studies by demonstrating that the association between self-esteem and NE inertia replicates (a) in a highly controlled laboratory context and (b) while controlling for between-person differences in the mean level and variability of NE.

### Negative Indicators of Well-Being

In contrast to our findings for positive indicators of well-being (see above), all four negative indicators of well-being showed consistent positive associations with both NE and PE inertia across studies and analytic methods (see meta-analysis in **Table 4**). However, in line with Houben et al.’s (2015) meta-analysis, NE inertia was more strongly associated with negative indicators of well-being than PE inertia in the current studies. Extending upon previous research, we found that associations between negative indicators of well-being and NE inertia tended to become stronger, on average, when examining standardized NE scores. For instance, the average correlation between trait rumination and NE inertia across studies was 0.19 and this increased to 0.26 after standardizing NE ratings. In contrast, associations with PE inertia became slightly weaker when examining standardized PE ratings.

## Discussion

The current studies aimed to replicate and extend upon previous research that has linked higher emotional inertia with reduced well-being. Across two studies using a controlled laboratory paradigm to assess individual differences in emotion dynamics, we found consistent evidence for an association between higher emotional inertia and lower well-being. In particular, higher inertia of NE was most consistently and strongly associated with higher scores on negative indicators of well-being (i.e., depressive symptoms, trait rumination, NA and neuroticism). To a lesser extent, negative indicators of well-being were also related to greater inertia of PE. Finally, higher NE inertia was associated with lower levels of self-esteem. These findings are broadly consistent with a recent meta-analysis of the literature on emotion dynamics (Houben et al., 2015). However, the current findings go beyond previous research by helping to address a number of unresolved questions regarding the association between emotional inertia and well-being. We discuss each of these, in turn, below.

### Endogenous versus Exogenous Influences

Previous research has largely assessed emotional inertia in naturalistic contexts (e.g., using experience sampling), leaving open the possibility that individual differences in inertia are driven by external/contextual factors. In contrast, the current studies assessed emotional inertia using a controlled laboratory paradigm, in which all participants responded to a standardized sequence of emotional stimuli (i.e., films). This methodology allows us to rule out the influence of environmental factors on individual differences in emotional inertia. Rather, in the current studies, higher levels of emotional inertia reflect endogenously driven emotional inflexibility. We can therefore conclude, for instance, that the association between NE inertia and greater depressive symptoms observed in the current studies must be due to alterations in emotional responding (e.g., impaired recovery) rather than to differential exposure to emotional events (see also, Koval et al., 2015a).

### Inertia of Positive versus Negative Emotions

The current studies also extended upon previous research by examining how inertia of both negative and positive feelings relates to well-being. Although PE inertia was also significantly associated with lower well-being, this association was weaker than for NE inertia, in line with a recent meta-analysis (Houben et al., 2015). Thus, heightened NE inertia appears to be more predictive of well-being than PE inertia. Nevertheless, the current findings do not support the view that “happiness is best kept stable” (Gruber et al., 2013). In contrast to Gruber et al.’s (2013) findings, we found that greater moment-to-moment stability of PE was significantly correlated with higher depressive symptoms, rumination, trait NA, neuroticism, and lower self-esteem. We note that these divergent findings may be due to the use of different indices of emotion dynamics. Whereas the current studies focused on the autoregressive slope of emotions, Gruber et al. (2013) measured emotional variability and instability using the *SD* and MSSD, respectively (see Koval et al., 2013). Another important issue to consider when interpreting indices of PE and NE dynamics is the timescale at which emotions are assessed (Hollenstein et al., 2013; Hollenstein, 2015). For instance, higher levels of emotional variability or instability across hours or days may reflect dysregulated emotions, whereas greater variablity at shorter timescales (e.g., seconds, minutes) may be adaptive. Remarkably, however, studies of emotional inertia at various timescales from seconds (Kuppens et al., 2012) to minutes or hours (Koval et al., 2013) and even days (Brose et al., 2014) have consistently linked higher inertia (especially of NE) with lower well-being. Finally, elevated PE inertia may be more pronouced in specific clinical groups, such as among individuals with bipolar disorder who may experience perseveratively high levels of PE across contexts (Gruber, 2011).

### Positive versus Negative Indicators of Well-Being

The current studies also went beyond previous research by assessing a broader range of well-being measures, including both negative and positive indicators. Whereas all negative indicators of well-being were significantly associated with higher emotional inertia, among positive well-being indicators only self-esteem showed consistent negative associations with NE inertia across studies and analytic methods. In contrast, PA, extraversion and satisfaction with life were less strongly and consistently related to emotional inertia. Thus, based on the current findings, it would be fair to conclude that emotional inertia, particularly of NE, is associated with higher levels of maladjustment and lower self-esteem but not with positive indicators of well-being more broadly. However, it may be premature to conclude that emotional inertia is not related to positive indicators of well-being until different kinds of positive psychological functioning (e.g., social connectedness, personal strengths, academic and job satisfaction, relationship quality) are studied in relation to emotional inertia. It is noteworthy, however, that each of the well-being measures that was consistently associated with higher emotional inertia in the current studies has been identified as a vulnerability factor for clinical depression, including elevated depressive symptoms (Klein et al., 2013), higher neuroticism (Lahey, 2009), the tendency to habitually ruminate (Nolen-Hoeksema et al., 2008), higher levels of trait NA (Watson et al., 1988a) and lower levels of self-esteem (Sowislo and Orth, 2013). Thus, the current findings support recent evidence suggesting that emotional inertia may be an early warning sign for depression (van de Leemput et al., 2014). However, studies using longitudinal measurement-burst designs (Röcke and Brose, 2013) would be needed to conclusively demonstrate that emotional inertia predicts the development of depression over time.

### Emotional Inertia versus Emotional Variability

Finally, an important contribution of the current studies is that we estimated associations between emotional inertia and well-being based on both raw and within-person standardized emotion ratings. Analyses based on standardized scores ensure that mean levels and variability (i.e., *SD*) of emotions are held constant across individuals, allowing for a purer estimation of the relationship betweeen temporal dependency (i.e., autocorrelation) of emotions and well-being. In particular, given that numerous studies have linked higher emotional variability with reduced well-being (Houben et al., 2015), it is important to establish the distinct contribution of emotional inertia. We note that in the current studies associations between NE inertia and well-being remained significant (even becoming stronger) when analyzing standardized NE ratings. This corroborates our previous claims that higher NE inertia is not merely an artifact of greater variability or mean level of NE (Koval et al., 2013). However, our findings differed somewhat for PE inertia: associations betwen PE inertia and well-being either remained similar or became slightly weaker when controlling for individual differences in mean level and variability of emotions. Clearly, further research is needed to properly disentangle the roles of mean level, variability and inertia of PE and NE in relation to well-being. Novel statistical models that allow for simultaneous estimation of the mean, variability and temporal dependency of a process are promising in this regard (Wang et al., 2012; Jongerling et al., 2015).

### Limitations and Future Directions

The current studies are characterized by a number of limitations, suggesting avenues for future research. First, the use of predominantly female undergraduate samples limits the generalizability of the current findings, especially given gender (Nolen-Hoeksema, 2012) and age (Urry and Gross, 2010) differences in emotion regulation processes, which play a central role in shaping emotion dynamics. Thus, an important direction for future research is to explore whether emotional inertia shows similar associations with well-being across different age and gender groups. Second, while the current studies support recent suggestions that emotional inertia may be a marker of depression vulnerability, it remains to be seen whether emotional inertia can equally be considered a vulnerability factor for other psychopathologies, such as anxiety, psychosis, or various personality disorders (see Houben et al., 2015). In particular, further research with clinical samples is warranted to clarify whether heightened inertia predicts psychological functioning among individuals with current or remitted psychopathology (Höhn et al., 2013; van de Leemput et al., 2014). Finally, the current studies examined only the subjective feeling component of emotions. Given that emotions are thought to involve loosely coupled changes in behavior, physiology, and subjective experience (Scherer, 2009), there is a need to study the temporal dynamics of emotions as multi-componential processes. Preliminary evidence suggests that various emotion components may show distinct patterns of temporal dynamics (Koval et al., 2015b). However, additional research is required to properly characterize how different components of emotions unfold and relate to each other over time. Despite these limitations, the current studies make an important contribution to the literature on emotion dynamics by examining how inertia of both PE and NE, assessed under controlled laboratory conditions, relates to a broad range of well-being measures, while controlling for individual differences in the mean level and variability of emotions.

## Author Contributions

PKo, PKu, and SS conceived and designed the studies. PKo collected the data. PKo analyzed the data and drafted the manuscript, and PKu and SS provided critical revisions and approved the final version of the manuscript.

## Conflict of Interest Statement

The authors declare that the research was conducted in the absence of any commercial or financial relationships that could be construed as a potential conflict of interest.

## Footnotes

1 The terms ‘affect dynamics’ and ‘emotion dynamics’ are often used interchangeably in the literature (Kuppens, 2015).

2 An alternative label for this broad construct might be *psychological functioning*.

3 Hamaker and Grasman (2015) recommend person-mean centering the lagged predictor when examining associations between the autoregressive slope (i.e., emotional inertia) and Level-2 predictors (e.g., well-being), but not when estimating the average level of emotional inertia (see below).

4 Two participants in Study 1 showed no variation in their NE ratings in the film-task (i.e., *SD* = 0), and were therefore excluded from analyses using standardized NE ratings.
